# Searching the Link for Better Therapeutic Combination: The Case of Tumor Cells Migration Pattern and Modality of Immunosuppression Induction at the Metastatic Site

**DOI:** 10.7759/cureus.7353

**Published:** 2020-03-21

**Authors:** Mohamed Islam Delma, Chiara Riganti

**Affiliations:** 1 Internal Medicine, Colonel Chaabani Hospital, El Menia, DZA; 2 Oncology, University of Torino, Torino, ITA

**Keywords:** metastasis, myeloid-derived suppressor cells, drug combinations, integrins, n-cadherin

## Abstract

Cancer is a disease characterized by its high morbidity and mortality, mainly due to its metastatic ability. Metastasis is a multi-step process beginning with detachment of tumor cells from the primary tumor and leading ultimately to the establishment of a new tumoral site. This cascade includes intravascular migration of tumor cells either individually or collectively and the expansion of cancer cells at metastatic sites that is dependent on certain conditions such as an immunosuppressive environment. In this paper, blockers of tumor cell migration and suppressors of immunotolerance at metastatic sites are reviewed as an illustration of early and later phases intervention, respectively. A combination of these two therapeutics will be advocated based on the proposition of correlation between the pattern of tumor cell migration and the mechanism of immunotolerance induction. By extension, the ''delayed complementarity'' will be introduced as an approach to formulate new anticancer drug combinations.

## Introduction and background

Metastasis is the ensemble of steps that some tumor cells follow to migrate from a tumor site to another distant one [1]. First, these cells have to invade through the extracellular matrix (ECM) to reach a vascular vessel. After they penetrate the vessel wall, they migrate through the circulatory system either individually as circulating tumor cells (CTC) or collectively as circulating tumor microemboli (CTM) [2]. Once they have reached the target site, they exit the vascular system by extravasation, cut across the ECM to reach their new "home". The development of the metastatic site reflects the survival and proliferation of tumor cells in the hostile new environment that is facilitated by establishing some favorable conditions such as immunotolerance towards these cells and a vasculature system for blood supply. Each step of the cascade is a potential therapeutic target to block the metastatic process. As an illustration, inhibitors of tumor cell migration and immunotolerance suppressors will be reviewed in the next section.

## Review

Inhibition of tumor cell migration

Collective and individual tumor cell migration inhibitors share a common target which is cell motility, but they have other specific ones such as intercellular adhesion for collective migration blockers. Of note, collective migration inhibitors act from the initial step of invasion at the primary site, given that most probably the cells are clustering at this point [2]. At a molecular level, the principal actors targeted are Rho GTPases, N-cadherin, and integrins. Rho GTPases are members of Rho family proteins that act as intracellular transducers mediating the organization of various types of actin filaments, thus playing an important role in cells migration. Rho GTPases include many molecules such as RhoA, Rac1, and Cdc42 and have multiple effectors mainly the Rho-associated coiled coil-containing protein kinases (ROCKs) [3]. In addition to their permissive role in tumor cell migration, Rho GTPases have specific effects on collective migration by maintaining the front-rear polarity of cell clusters and the stability of cell-cell junction [4]. Rho GTPases pathway blockers target either Rho GTPases or their downstream effectors. GGTI-2418 is an inhibitor targeting a post-translational modification required for the function of Rho proteins. It has progressed to clinical phase I, but the study was terminated prematurely based on sponsor decision [5]. As for ROCK inhibitors, a drug labeled AT13148 has completed the phase I, but the results have not been reported yet. Another approach for targeting this pathway is through nonspecific commonly used drugs that have shown an inhibitory effect on the Rho GTPases pathway, such as statins and the phytochemical agent known as rocaglamide [3,6].

Integrins are transmembrane heterodimers formed by α and β subunits that intervene in collective cell migration by maintaining cells cluster’s cohesion via homophilic integrin-integrin cell adhesion, and the leader-rear polarity through cytoskeletal coordination [7]. The α5β1 inhibitor volociximab has progressed to the phase II trial and had demonstrated an acceptable safety profile. Despite the encouraging preclinical outcomes of β1 integrins blockade, the clinical benefice was still insufficient [8]. Some possible explanations of the limited outcomes have been discussed in detail by Alday-Parejo and colleagues, such as the subtypes of integrins that have been targeted, the methods of blockade used, in addition to some concerns about preclinical models selected and the designs of clinical trials [9]. 

Another molecule implicated in collective migration is N-cadherin. This transmembrane protein mediating cell-cell adhesion is expressed by different cells such as endothelial and neural ones, but also some tumor cells [10]. It is upregulated at the invasive front via the process of epithelial to mesenchymal transition that precedes tumor cell detachment and invasion, providing an adherence between the migrating cells. ADH-1, a cyclic pentapeptide, is the first N-cadherin inhibitor to be tested in the clinical setting. In phase I, it had shown an acceptable safety profile with some clinical efficacity [11]. More data would be obtained from recently completed phase II studies. Another class of N-cadherin inhibitors is monoclonal antibodies that have demonstrated suppressive effects on proliferation, invasion, and metastasis in vitro and in vivo [12]. Given that N-cadherin is cleaved by proteases such as the disintegrin and metalloprotease 10 (ADAM10), targeting N-cadherin through ADAM10 agonists would be rational [13]. Unfortunately, ADAM 10 has nonspecific cleavage activity that may induce activation of some protumorigenic molecules [14]. This could be the reason for not including ADAM10 agonists in cancer clinical trials, although its use in other conditions such as Alzheimer's disease [14]. However, some commonly used anticancer drugs had proven to have ADAM10 agonist effects such as the synthetic retinoid Am80 (tamibarotene) and the natural phenol, resveratrol. This incites for an extensive evaluation of ADAM10 agonist effects on cancer. 

Inhibition of immunomodulation

Immunotolerance provides a favorable environment for migratory tumor cells to develop at metastatic sites. It may be achieved by myeloid-derived suppressor cells (MDSCs), even before the arrival of metastatic cells. MDSCs are a heterogeneous population of immature myeloid cells that suppress innate and adaptive immunity [15]. This effect is accomplished through different mechanisms such as the production of reactive nitrogen and oxygen species, depletion of key nutrients factors for T cells such as L-arginine and L-tryptophan, limiting immunocompetent cells homing, induction of regulatory T cells, and production of immunosuppressive cytokines [16]. Tumor cells could also induce immunomodulation as they arrive at the metastatic site via immunosuppressive checkpoints molecules expressed on their surface, such as PDL1, TIM-3, B7-H3, B7-H1, CD47, and immunomodulatory secreted factors such as IL-8, IL-6, and TGFB [15]. The focus in this paper will be on inhibitors of MDSC-induced immunotolerance.

Broadly, MDSC may be neutralized by at least three different approaches [17]. We can either block their immunosuppressive effect, limit their homing to metastatic sites, or reduce their numbers. Suppression of the immunomodulatory effect may be achieved by different drugs. Some of them are commonly used such as tadalafil, and others are under experimentation such as STAT3 inhibitors. Tadalafil is a phosphodiesterase type 5 (5PDE) inhibitor that had shown amelioration of clinical outcomes in patients with head and neck squamous cell carcinoma, and melanoma [18-19]. These results are due in part to inhibition of MDSC activity through suppression of NO arg-1 pathways [18]. By the same mechanism, the class I histone deacetylase inhibitor entinostat had shown an inhibitory effect on MDSC function in preclinical models and has progressed to phase II trials as a monotherapy and in combination [20]. One of these trials had already published the results, showing a 24% rate of disease control with entinostat [21]. STAT3 had shown an important role in MDSC immunomodulation, making it a therapeutic target [22]. There are at least six major classes of STAT3 inhibitors [23]. Napabucasin, an inhibitor of transcription of STAT3 downstream genes, is actually the only one that had progressed to phase III clinical trials. It had shown prolongation of overall survival in patients with positive pSTAT3 advanced colorectal cancer [24]. Antagonism of leukocyte immunoglobulin-like receptor B, a myeloid receptor intervening in MDSC differentiation and tumor immune tolerance, seems also a promising approach as it has shown a reprogramming effect on MDSC toward an antitumoral phenotype [25-26].

Limiting MDSC recruitment to pre-metastatic niches could be achieved by targeting chemokine/chemokine receptors involved, such as CCL2/CXCR2 or CCR5. CCR5 inhibitors had shown antitumoral effects on preclinical models and one of them, maraviroc, had been already approved for clinical use in HIV, facilitating thus the realization of a pilot clinical trial in patients with advanced metastatic colorectal carcinoma [27]. The drug was well-tolerated, with evident antitumoral activity at histologic level, and some partial clinical response. As for CCL2/CXCR2 axis blockade, AZD5069 and SX-682 are two molecules that are currently tested in phase I and II clinical trials [28]. Lastly, some chemotherapeutic drugs have shown an MDSC reduction effect such as the anti-CD38 antibody daratumumab that has been approved by the FDA for multiple myeloma. Studies have shown a depleting effect of daratumumab on MDSC, given their expression of CD38. The list also includes all-trans retinoic acid (ATRA), gemcitabine, and 5-fluorouracil. Their effects are exercised through different mechanisms such as apoptosis and induction of differentiation of MDSC into mature myeloid cells like DC or macrophages [17].

**Figure 1:**
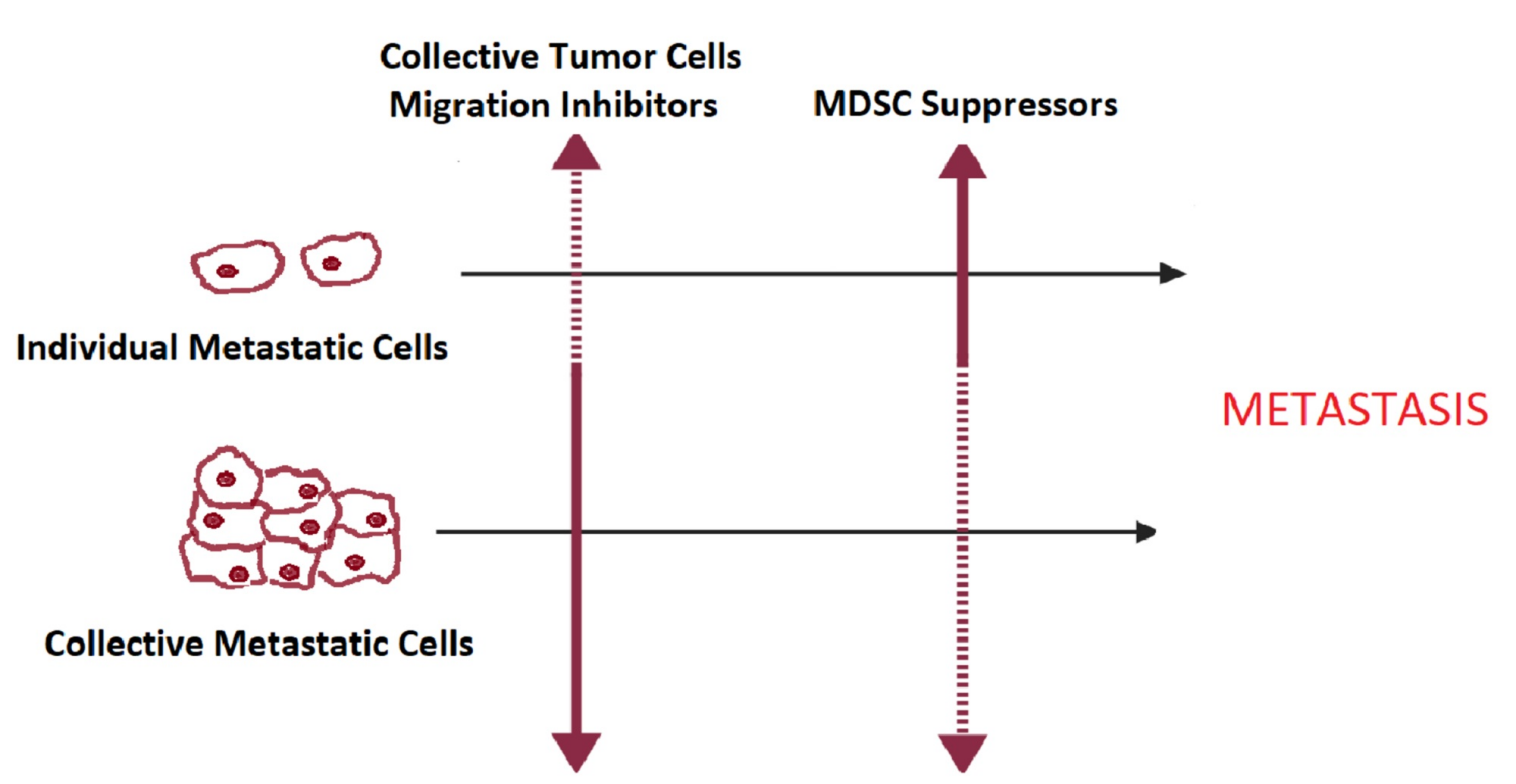
Schematic illustration of delayed complementarity between collective tumor cell migration inhibitors and MDSC suppressors MDSC, myeloid-derived suppressor cells

A general remark about tumor cell migration blockers and tumor immunotolerance inhibitors is that they both have at best partial response as monotherapy. So, using these inhibitors in combination with other molecules seems to be a must. The combination is classically achieved by addition of commonly used chemotherapeutic drugs. In a model of rat sarcoma, Ten Hagen *et al*. had shown a synergism between αVβ3/αVβ5 integrin inhibitor cilengitide and the alkylating agent melphalan [29]. However, the association between cilengitide and temozolomide chemoradiotherapy did not ameliorate the outcome in a multicenter phase 3 trial [30]. The selection of drugs may be also based on physiopathological approach. Given that each metastatic step may be accomplished by different mechanisms that may not all be suppressed by one inhibitor, combining two inhibitors of the same metastatic step but acting on different mechanisms is rational. An example extensively tested in experimental models is the association between MDSC-targeting drugs and immune checkpoint inhibitors. This combo had shown encouraging outcomes and is adopted by many ongoing clinical trials [31]. In this paper, an additional physiopathological approach is proposed based on ''delayed complementarity''. It consists of suppressing metastatic cells that escape a given inhibitor by a second one acting at a later phase of metastasis. To be applicable, this approach needs a certain degree of predictability between the two phases that are targeted. That means that tumor cells following a pathway that is not blocked by the first inhibitor are expected to depend at the later phase on a mechanism repressible by the second agent. So, can this approach be used to realize a combination between tumor cells migration inhibitors and immunotolerance suppressors? To have an answer, we should first find out if there is a correlation between the type of migration and the modality of immunotolerance induction at the metastatic sites. Theoretically, if the definitions and characteristics are taken into account, there would be a link. In individual cell migration, the preponderant mechanism to induce immunotolerance would be via MDSC, as it would be less probable for single tumor cells to induce important changes in the immune microenvironment as they arrive. As for collective migrating tumor cells, tumor-induced immunotolerance would also take part in the process, given the number of tumor cells migrating simultaneously, and eventually, the presence of cells with an immunosuppressive ability such as tumor-associated fibroblast,s within the microemboli [32-33]. This implies that the association between tumor cell migration inhibitors and immune tolerance suppressors would be possible. An efficient combination of these therapeutics would be achieved by targeting the collective tumor cell migration and MDSC, given that individual migratory cells that may escape the collective migration inhibitor would rely later on MDSC for immunotolerance induction (Figure 1).

Finally, some limitations of the delayed complementarity approach should be taken into consideration. It is expected that the success of such a combination would depend heavily on the suppressive ability of each drug, and so developing therapeutics with greater efficacity is the challenge to optimize the results. Also, the association of tumor cell migration inhibitors and immunotolerance blockers is targeting mainly the metastatic process. If it demonstrates satisfactory results, therapeutic interventions targeting the primary tumoral site should be considered. Of note, this illustrated combination should be introduced at an earlier phase of cancer progression, as it is acting on the prevention of metastatic site formation. 

## Conclusions

In the absence of radical treatment for cancer disease, association of available drugs is a valuable method to ameliorate the clinical outcomes. The combination of therapeutics targeting different phases of cancer progression that have some predictable connections between them is the basis of delayed complementarity approach. If validated, this would be an additional option to formulate more effective anticancer drug combinations. 
